# Real-Time Surveillance of Infectious Diseases and Other Health Conditions During Iraq’s Arbaeenia Mass Gathering: Cross-Sectional Study

**DOI:** 10.2196/14510

**Published:** 2019-10-04

**Authors:** Faris Lami, Inam Hameed, Abdul Wahhab Jewad, Yousef Khader, Mirwais Amiri

**Affiliations:** 1 Department of Community and Family Medicine College of Medicine University of Baghdad Baghdad Iraq; 2 Karbala Directorate of Health Iraq Ministry of Health Karbala Iraq; 3 Najaf Directorate of Health Iraq Ministry of Health Najaf Iraq; 4 Faculty of Medicine Jordan University of Science and Technology Irbid Jordan; 5 Center of Excellence for Applied Epidemiology, Global Health Development Amman Jordan

**Keywords:** mass gathering, Arbaeenia, surveillance, Iraq

## Abstract

**Background:**

The most common religious mass gatherings in the Middle East are the Hajj at Mecca in Saudi Arabia, which occurs annually, and the Arbaeenia in Karbala. The importance of developing public health surveillance systems for mass gatherings has been previously emphasized in other reports.

**Objective:**

This study aimed to describe the common illnesses and health conditions affecting people during the Arbaeenia mass gathering in Iraq in 2016.

**Methods:**

A total of 60 data collectors took part in the field data collection over a period of 11 days, from November 12, 2016 to November 22, 2016. Data were collected from 20 health outlets along the major route from Najaf to Karbala (10 health facilities in each governorate). Two digital forms, the Health Facility Survey and the Case Survey, were used for data collection.

**Results:**

A total of 41,689 patients (33.3% female and 66.7% male) visited the 20 health care facilities over a period of 11 days from November 12, 2016 to November 22, 2016. More than three quarters of patients (77.5%; n=32,309) were between 20-59 years of age, more than half of patients were mainly from Iraq (56.5%; n=23,554), and about 38.9% (n=16,217) were from Iran. Patients in this study visited these health care facilities and presented with one or more conditions. Of a total 41,689 patients, 58.5% (n=24,398) had acute or infectious conditions and symptoms, 33.1% (n=13,799) had chronic conditions, 23.9% (n=9974) had traumas or injuries, 28.2% (n=11,762) had joint pain related to walking long distances, and 0.3% (n=133) had chronic dermatologic conditions.

**Conclusions:**

The Arbaeenia mass gathering in 2016 exerted a high burden on the Iraqi health care system. Therefore, efforts must be made both before and during the event to ensure preparedness, proper management, and control of different conditions.

## Introduction

The World Health Organization (WHO) defines mass gatherings as "events attended by a number of people sufficient to strain the planning and response resources of a community, state or nation[1]."

Such occurrences put a strain on local resources like food, drinks, accommodations, and health care, and they can pose a health risk to the population. Masses gather for many reasons, including religious activities, festivals, sporting events, and political rallies, among other reasons. The most common religious mass gatherings in the Middle East are the Hajj at Mecca in Saudi Arabia, which occurs annually, and the Arbaeenia in Karbala.

Iraq hosts one of the largest religious mass gatherings in the world. Every year, people from around the world visit Karbala in Iraq to observe the death anniversary of Imam Hussain ibn Ali, who was a revered Muslim leader. Millions of people gather at the Arbaeenia to mark this important event. The approximate number of visitors increased from 3 million people in 2003 to 25 million in 2016, with about 20% coming from countries other than Iraq [2]. According to 2014 estimates, Karbala City, where the gathering is hosted, has a local population of about 1.1 million individuals in an area of approximately 43.7 km^2^ [3,4].

Like other types of mass gatherings, the large number of individuals at this event poses communicable disease health risks and strains the limited health care infrastructure and services of the area. The risk of transmission and importing infectious diseases is high during the mass gathering, due to poor border crossing security [5]. The growing number of individuals who attend the event annually, the changing dates of the anniversary, and the short duration of the event highlight the importance of national and local authorities having preparedness plans in place and the resources to effectively manage the gathering. However, services for the Arbaeenia mass gathering, including health care, are not well developed in Iraq, thus hampering the ability of workers to both stop the spread of pathogens and detect and respond to outbreaks in a timely manner [6].

Although this mass gathering has complex challenges and is associated with an elevated risk for illnesses, there have been few studies done on the burden of religious mass gatherings on Iraq’s local health care resources. The importance of developing public health surveillance systems in mass gatherings was previously emphasized in other reports [7]. Therefore, the Eastern Mediterranean Public Health Network (EMPHNET) collaborated with the Iraq Field Epidemiology Training Program (FETP) and the Iraq Ministry of Health to apply real-time surveillance to several common health conditions (infectious or acute conditions, injuries, and chronic diseases) during the Arbaeenia mass gathering in Iraq between November 12, 2016 and November 22, 2016. This study aims to describe the common illnesses and health conditions affecting people during the Arbaeenia mass gathering in Iraq in 2016.

## Methods

**Figure 1:**
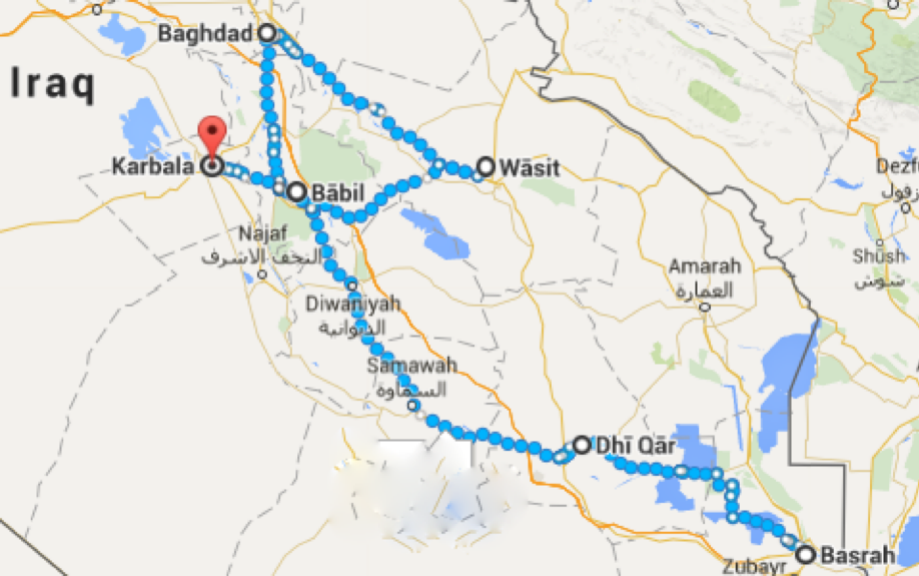
Map indicating the major routes for visitors travelling on foot during the annual Arbaeenia religious mass gathering.

A total of 60 data collectors took part in the field data collection over a period of 11 days (between November 12, 2016 and November 22, 2016). Data were collected from 20 health care facilities along the major route from Najaf to Karbala (10 health care facilities in each governorate). All patients that visited the facilities over this period of 11 days were interviewed. Figure 1 shows the map indicating the major routes for visitors travelling on foot during the annual mass gathering. EMPHNET conducted one set of training for the team of survey supervisors, which was followed by two subsequent sets of training by supervisors for data collectors in both Najaf and Karbala. The team of data collectors were overseen and supported technically by four surveillance supervisors (two in each governorate).

A total of 20 tablets were provided to the field team (1 for each health care facility), and the Data4Action platform was used to put the survey tool on the tablets. The information technology team at EMPHNET, with input from the technical team, worked on digitizing the survey tool for real-time surveillance. Two forms (the Health Facility Survey and the Case Survey) were published online at 12:00 midnight on November 12, and the official surveillance activity (field data entry) started at 8:00 AM on November 12, 2016. For data that needed to be entered for testing purposes (eg, in case of the tablets malfunctioning), the data collectors were told to put [TEST] in response to the very last question on the last page of the Case Survey Form before submission. Those TEST case entries were later dropped from the analysis.

Given the simplicity of the survey form, the duration of each case interview ranged from as short as 15 seconds to a few minutes. However, the average duration of the interviews was about 1 minute and 21.3 seconds. This is a valuable piece of information for planning any similar events on an even larger scale.

The case form included information on facility identification (ID), medical staff ID, case ID, age of patients in years, gender, and educational level. The health conditions or complaints (Symptomatic Surveillance) included acute infectious conditions like acute watery diarrhea, acute bloody diarrhea, vomiting with or without diarrhea, fever and cough or flu, fever and bleeding tendency, and fever and rash. Dermatological conditions including itching and skin rash without fever were part of the form. We included conditions related to trauma and walking for long distances. like wounds, blisters, accidental injuries (fracture, car, trolley, or araba accident, overcrowding, falling, etc), and joint pain. Health complaints related to chronic conditions like blood pressure and blood glucose problems, ischemic heart pain or symptoms, and asthma symptoms were also included.

The two teams of surveillance supervisors (in both Najaf and Karbala) were told to provide regular daily briefings to the technical support team. These daily briefs were sent at the end of each day (usually after 5:00 PM) via email. Any major highlights of the day were included in these briefs. These highlights included, but were not limited to, the following: overcrowding of cases in any of the health care facilities during that specific day, issues faced by data collectors alongside their solutions from the supervisors’ team, specific challenges in the field, how support was sought from the central or technical team, and so on.

Real-time access to the collected online data was provided to the surveillance supervising team in Iraq. This enabled the technical staff to watch the data collection in real time and take any actions in case the need arose. The field data collection officially ended at midnight on November 21, although some cases were recorded during the early morning hours of November 22.

Extensive algorithms and carefully structured logical formulas were used throughout the database to clean, validate, process, and prepare the data for analysis. All implausible values were dropped during the data cleaning process. Global Positioning System coordinates of all locations (such as countries of origin, or governorate in case they came from inside Iraq) were added to the dataset to enable visualization of the spatial data on map. For this study, data were analyzed using SPSS (IBM Corp, Armonk) and described using percentages and graphs. Excel (Microsoft, Redmond) was used to develop the graphs.

## Results

### Patients’ Characteristics

A total of 41,689 patients (33.3% female and 66.7% male) attended the 20 health care facilities over a period of 11 days between November 12, 2016 and November 22, 2016. The total number of patients, according to each facility, ranged from 383 to 4346, showing that some health facilities recorded considerably more cases than the others due to their locations. More than three quarters of patients (77.5%; n=32,309) were between 20-59 years of age. A total of 41,482 patients had information on their nationality. More than half of patients were from Iraq (56.5%; n=23,554), 38.9% (n=16,217) were from Iran, and 1.4% (n=584) were from Bahrain, followed by small percentages of people from Lebanon, Saudi Arabia, Kuwait, Pakistan, India, Oman, and Afghanistan, and a total of 45 cases were recorded from 21 other countries. Among the recorded 23,444 Iraqis, the larget proportion of people (65.5%; n=15,356) came from Basra, Najaf, Dhi Qar, and Maysan governorates.

### Main Conditions and Complaints at Presentation

#### Overview

Patients visited the health care facilities for one or more conditions. Of a total of 41,689 patients, 58.5% (n=24,398) had acute or infectious conditions and symptoms, 33.1% (n=13,799) had chronic conditions, 23.9% (n=9974) had traumas or injuries, 28.2% (n=11,762) had joint pain related to walking long distances, and 0.3% (n=133) had chronic dermatologic conditions.

#### Acute or Infectious Conditions and Symptoms

There were 24,398 patients with acute or infectious conditions and symptoms during the event. About two thirds of those patients (68.5%; n=16,711) had fever and cough or flu. Other major categories were food poisoning cases with vomiting and diarrhea (6.3%; n=1526), acute dermatological conditions including itching and skin rash without fever (6.0%; n=1465), acute watery diarrhea (5.0%; n=1214), and fever and rash (2.6%; n=624). Table 1 shows the distribution of acute or infectious conditions and symptoms among patients who attended health care facilities for these conditions during Iraq’s Arbaeenia mass gathering in 2016.

**Table 1:** The distribution of acute or infectious conditions among patients who attended health care facilities for these conditions during Iraq’s Arbaeenia Mass Gathering, November 2016.

Acute conditions or symptoms	Count, n (%)
Fever and cough or flu	16,711 (68.49)
Food poisoning	1526 (6.25)
Acute bloody diarrhea	1470 (6.03)
Acute dermatological conditions	1465 (6.01)
Acute watery diarrhea	1214 (4.98)
Fever and bleeding tendency	982 (4.03)
Fever and rash	624 (2.56)
Other acute illnesses	2610 (10.69)

#### Chronic Conditions

The reasons for visiting health care facilities among those with chronic conditions included problems such as hypertension (55.3%), diabetes mellitus (26.1%), asthma (11.8%), ischemic heart pain or symptoms (3.5%), and other chronic illnesses.

#### Traumas and Injuries

Out of the total 9974 cases of injuries, the most prevalent complaints reported were blisters due to walking long distances (68.5%; n=6836), wounds (26.2%; n=2614), accidental injuries (4.3%; n=424), and fractures (1%; n=100).

## Discussion

### Main Findings

This study showed that the Arbaeenia mass gathering placed tremendous pressure on local health care resources and extra burden on health care facilities in Iraq. A total of 41,689 patients visited the 20 health care facilities during a period of 11 days throughout the mass gathering. Of those, 58.5% (n=24,398) had acute or infectious conditions, 33.1% (n=13,799) had chronic conditions, 23.9% (n=9974) had traumas or injuries, 28.2% (n=11,762) had joint pain related to walking long distances, and 0.3% (n=133) had dermatologic conditions.

Mass gatherings create favorable conditions for infectious disease transmission. This study showed that more than half (58.5%) of patients presented with acute symptoms or infectious conditions. Other studies have previously emphasized that there is an increased risk of infectious disease outbreaks during mass gatherings [8-10]. The risk of infectious disease outbreaks during mass gatherings is high because people are exposed to crowded and unhygienic environments, and most of the individuals preparing and serving food are not certified food handlers. Water and food safety, and compliance with health conditions, should be ensured both before and during the event. Food handlers should be trained and provided with working guidelines for safe food handling, and their practices should be inspected and monitored during the event.

Mass gatherings may also exacerbate noncommunicable diseases (NCDs) and chronic conditions, which may lead to emergencies and hospital admissions [11]. The incidence of severe acute cardiovascular events is more than doubled during mass gatherings for people exposed to intense stress [12-14]. High rates of morbidity and mortality from NCDs, including cardiovascular diseases, diabetes mellitus, and asthma, have previously been reported during the Hajj mass gatherings. Our study showed that 33.1% (n=13,799) of people that visited any health care facilities for chronic conditions had problems including hypertension, diabetes mellitus, asthma, and ischemic heart disease or symptoms. Walking long distances, changes in daily activities, and intense physical activity during mass gatherings may also worsen any preexisting conditions. Moreover, poor adherence to diet and medications could negatively affect NCD control, particularly asthma, hypertension, diabetes mellitus and ischemic heart diseases.

About one quarter (23.9%; n=9974) of patients presented at health care facilities with traumas or injuries. Most injuries occurring during mass gatherings are due to overcrowding [15,16]. Other important causes of injuries include some people practicing self-harm, like laceration of their scalp using sharp knives or other risky practices, because of cultural or religious beliefs [17]. Moreover, the journey to Karbala requires several days of walking, resulting in musculoskeletal pain and injuries. This might explain why our findings showed that one quarter of patients presented with joint pain. Given the high percentage of injuries during this event, the government should prepare traffic plans, enforce traffic safety and security laws, assign special streets for visitors that direct them away from traffic, raise visitors’ awareness regarding road safety, improve ambulance services, and train volunteers on emergency first aid and safe transportation of injured people.

### Conclusions

In conclusion, the Arbaeenia mass gathering in 2016 exerted a high burden on the health care system of Iraq. Therefore, efforts must be made both before and during the event to ensure preparedness, proper management, and control of different conditions. Such activities should include determining the essential services to be provided, determining the organizational structure of medical units and their human resources, creating a standard list for needs, essential medical equipment, and drugs, and inspecting the availability of the preceding requirements during mass gatherings. Moreover, other activities should ensure proper disposal of medical waste as well as infection control practices. Other recommendations include encouraging scientific research into the subject of mass gatherings to gather more information on their effects. Regarding a health surveillance and documentation system, there needs to be improvement in the process of documenting medical cases, which can be achieved by applying documented referral systems from medical units to other institutes, and documenting drug prescriptions, medical needs, and other logistic issues to calculate their financial costs. Furthermore, all districts that are visited by mass gathering attendees are urged to provide various forms of support (ie, financial, logistical, human resources), considering the density of the visitors. Finally, renewing the communicable syndromic surveillance system in medical units during mass gathering events, constructing complementary laboratories in major medical centers, providing transportation, and ensuring transportations of specimens to central laboratories are all recommended measures.

## Abbreviations

EMPHNET: Eastern Mediterranean Public Health Network
FETP: Field Epidemiology Training Program
ID: identification
NCD: noncommunicable disease
WHO: World Health Organization
